# Large Solitary Fibrous Tumor (SFT) of the penis- a case report and review of literature

**DOI:** 10.1186/s12894-023-01302-w

**Published:** 2023-08-02

**Authors:** Deep Kumar Jain, Himanshu Pandey, Sashank Saini, Sashikant Patne

**Affiliations:** 1 Uro-Oncology Division, Department of Surgical Oncology, Mahamana Pandit Madan Mohan Malviya Cancer Centre (MPMMCC) & Homi Bhabha Cancer Hospital (HBCH), Varanasi, India; 2 Department of Pathology, Mahamana Pandit Madan Mohan Malviya Cancer Centre (MPMMCC) & Homi Bhabha Cancer Hospital (HBCH), Varanasi, India

**Keywords:** Solitary Fibrous Tumor (SFT), Penile mass, Spindle cell tumors

## Abstract

**Background:**

Solitary fibrous tumors (SFTs) are very rare spindle cell neoplasms of mesenchymal origin with largely benign course of disease. Genital SFT’s can be managed providing excellent functional and psychological outcomes by timely intervention.

**Case presentation:**

We report the largest and possibly the second only reported case of penile SFT in a 34 year male presenting with a gradually increasing perineal mass with clinically normal appearing phallus. MRI revealed a 9.8 × 3.2 cm soft tissue mass arising from left corpora cavernosae, the mass was excised en-bloc via a perineal approach under spinal anaesthesia. Histopathology revealed spindle cell tumor embedded in myxohyaline stroma along with hyalinized vascular channels demonstrating IHC positivity for CD34 and STAT6. The patient is disease free post 2 years of resection with no sexual or urinary dysfunctions.

**Conclusion:**

Genital SFTs, although rare, should be considered in the differential diagnosis of well-circumscribed, painless, slow growing solid masses and histopathologists must be vigilant of its malignant characteristics.

## Background

The most common benign soft tissue tumors that affect the penis are vascular neoplasms, followed by tumors of neural, myoid and fibrous origin. Among reported cases, the most frequent malignant penile soft tissue tumors are Kaposi sarcoma and leiomyosarcoma [1]. Solitary Fibrous Tumors (SFT) are spindle cell neoplasms of mesenchymal origin previously also known as benign mesothelioma, localized mesothelioma, solitary fibrous mesothelioma, localized fibrous tumor and hemangiopericytoma due to the many overlapping gross and histological features. Although advances in histology and molecular genetics has helped differentiate STF’s from other soft tissue tumors, its clinical and histological course, management strategies and prognosis are largely based on case reports and few retrospective series. Majority SFT’s follow a benign clinical course however, as much as 20%—30% tumors reveal malignant features on gross and microscopic histopathological examination [2, 3]. Features suggesting malignant nature of the mass, as reported from various case series are, moderate to severe atypia, high cell density, mitotic activity ≥ 4 /high power field, margin infiltration, tumor necrosis [4–6] and tumor size ≥ 10cm [3, 7] or ≥ 10.5cm [8, 9]. Tumors with such features are known to recur and/or metastasize. It is important to note that no particular tumor location, imaging characteristics except tumor size, patient, environmental or dietary factors have been implicated in the occurrence or malignant behaviour of SFT’s thus far.

SFT’s, originally reported as pleural tumors by Klemperer and Rabin in 1931 [10], continue to pose a unique diagnostic and management challenge and have now been reported to arise from a wide range of anatomic sites, including penis [11–16]. To the best of our knowledge, this case is probably the largest penile SFT reported till date. The first case of penile SFT was reported by Castellani et al. in 2015 [17]. Although complete en-bloc resection remains the primary treatment for resectable diseases, with a favourable outcome, management of locally advanced, recurrent or metastatic disease continues to remain uncertain and probably involves a combination of chemotherapy and/or radiotherapy along with surgery when feasible. The unpredictable natural course of the disease coupled with rarity in its occurrence have dampened the development of standard management protocols, and thus, long term follow-up of patients being treated is essential in identifying key features and potential targets for surgical and systemic therapy respectively. Dilated and branching blood vessels, STAT6 protein overexpression and NAB2-STAT6 gene fusion are common to both hemangiopericytoma and SFT accounting for difficulties in differentiation and classification of these two entities [18, 19]. Thus the World Health Organization (WHO), in 2013, discontinued the term heamangiopericytoma and reclassified it as part of extra-pleural solitary fibrous tumors [20] and in 2016 introduced the term SFT-HMP [21]. Over the years, however, continued discovery and pooled research has led to further differentiation of hundreds of soft tissue masses including SFT. Furthermore, advances in histologic, molecular and genetic techniques have allowed more precise categorization and identification of soft tissue tumors. SFTs can broadly be classified into thoracic and extra-thoracic SFTs, however, they constitute a single entity when considering their biological behaviour including clinical and pathological features.

## Case presentation

### Clinical features

**Fig. 1:**
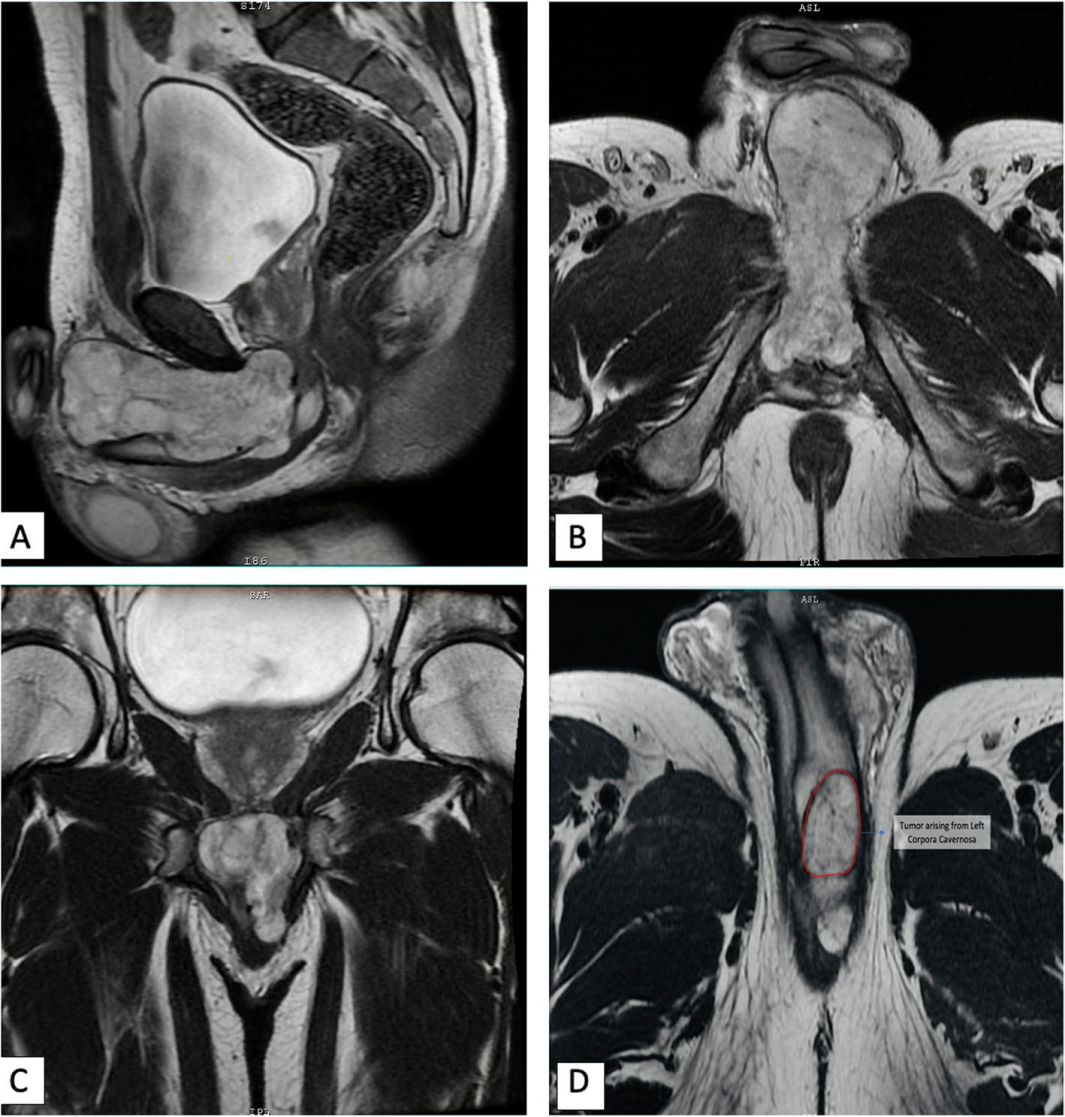
Contrast enhanced Pelvis MRI images (**A**)-Saggital, (**B**) & (**D**)- Axial and (**C**)-Coronal cuts

Thirty-four years male presented with complaints of painless swelling in perineum from 4 months. The swelling was gradually increasing in size extending towards base of scrotum and left inguinal region. Patient did not report any difficulty passing urine, hematuria or bleeding per-rectally. The mass was hard in consistency, non-tender, mobile and barely palpable per-rectally. Bilateral inguinal examination did not reveal any significant palpable lymphadenopathy. Urine microscopic examination reported colourless urine without significant white or red blood cells, no casts or bacteria. Magnetic resonance imaging (MRI) Pelvis revealed a 9.8 × 3.2 cm well-defined T2 heterogeneously hyperintense lesion arising from left corpora cavernosa at root of penis with exophytic component extending anteriorly for a distance of 9.6 cm (Fig. 1A-D). The exophytic component was seen to extend beyond the confines of tunica albugenia in left paracavernosal fat space. The corpus spongiosum appeared involved at the root of penis and right corpus carvernosum was compressed by the mass. Superiorly the lesion was abutting the apex of the prostate and urethra and distally it extends into the proximal most aspect of scrotal sac with displacement of left spermatic cord laterally with focal loss of intervening fat planes. Metastatic work-up with a 18-Fluorodeoxyglucose (FDG) Positron emission tomography (PET) contrast enhanced computer tomography (CECT) was negative for metastatic deposits. Urethrocystoscopic examination revealed mild bulge in bulbar urethra just distal to prostatic apex and external urethral sphincter region, however no intraluminal extension or infiltration was identified. The patient reported to us with an inguinal mass biopsy done elsewhere. The biopsy was reviewed by the consultant pathologist at our center and reported to show features of spindle cell tumor present in a myxo-hyaline stroma. The spindle cells were arranged in fascicles and scattered along with many vascular hyalinized channels. Significant nuclear atypia was not seen, mitotic figures were inconspicuous with focal area of necrosis. Immunohistochemistry (IHC) showed tumor cells to be positive for CD34 and negative for AE1/AE3, S-100, desmin, SMA, h-caldesmon and CD-31. The final impression in biopsy was of a benign spindle cell tumor, fibrohistocytic type. The patient’s sexual function assessment was done pre- and post-operatively using the International Index of Erectile Function (IIEF) questionnaire [22].

**Fig. 2:**
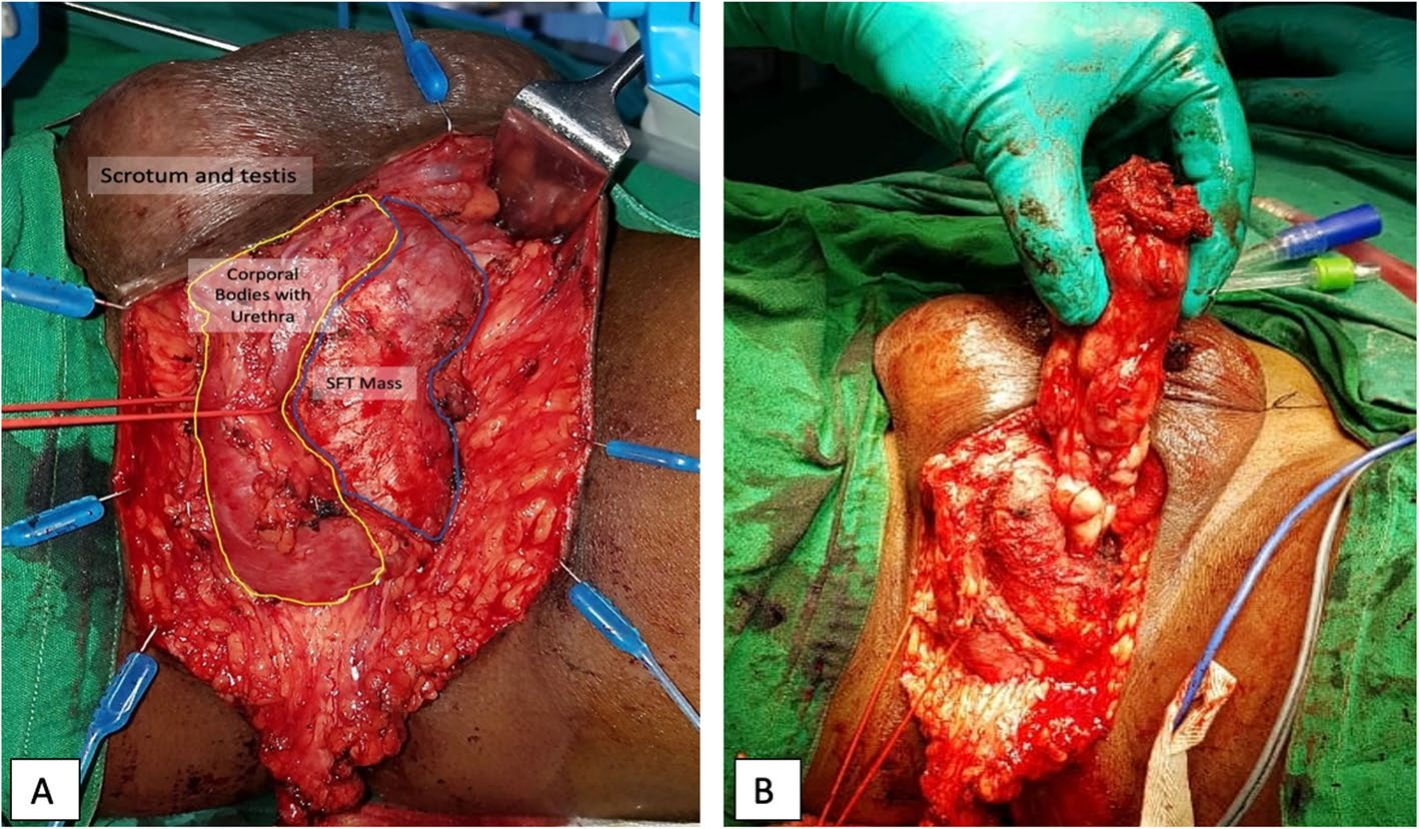
**A** Penile SFT mass and its relation to surrounding structures, **B** SFT mass post en-bloc excision attached only at its origin in the left corpora cavernosa

The patient underwent en-bloc excision of mass with consent for possible total penectomy and perineal urethrostomy. With the patient in lithotomy position and a 14 Fr Foley’s catheter insitu, an inverted u-shaped incision was taken with a vertical extension at the apex towards the base of the scrotum. The soft tissue mass was identified running along the left copora cavernosa ventrally and extending upto the base of the penis at level of the penile crus. The mass was removed en-bloc (Fig. 2A & B). A small defect in the left copora cavernosa, from where the mass seemed to be arising, was repaired with intermittent Polydiaxone 3.0 sutures, wound was closed in layers after placing a negative suction drain and a compression dressing with elastic adhesive tape was applied. The patient was discharged on post-op day two after removal of Foley’s catheter and drain.

### Post-op period

Postoperative period was largely uneventful, apart from mild post-op pain and swelling at the surgical site, no complications were encountered. The Negative suction drain was removed on 2^nd^ post-op day, patient was discharged and asked to review after 1 week for suture removal. Chordee or erectile dysfunction was not reported by the patient during postoperative follow-up. An abdominopelvic MRI was performed at 6 months, 1 year and 2 year after the surgery and no local recurrence or distant metastasis was identified. The scores recorded at baseline and at 6 months post-op for Erectile function were 30 and 29; Orgasmic function 10 and 9; Sexual desire 10 and 10; Intercourse satisfaction 15 and 12 and Overall satisfaction 10 and 8 respectively. At 1 year post-op, scores for Erectile function-29; Orgasmic function-10; Sexual desire-10; Intercourse satisfaction-14 and Overall satisfaction-10 were almost identical to the baseline scores and were persistent at 2 years follow-up.

### Histopathological features

**Fig. 3:**
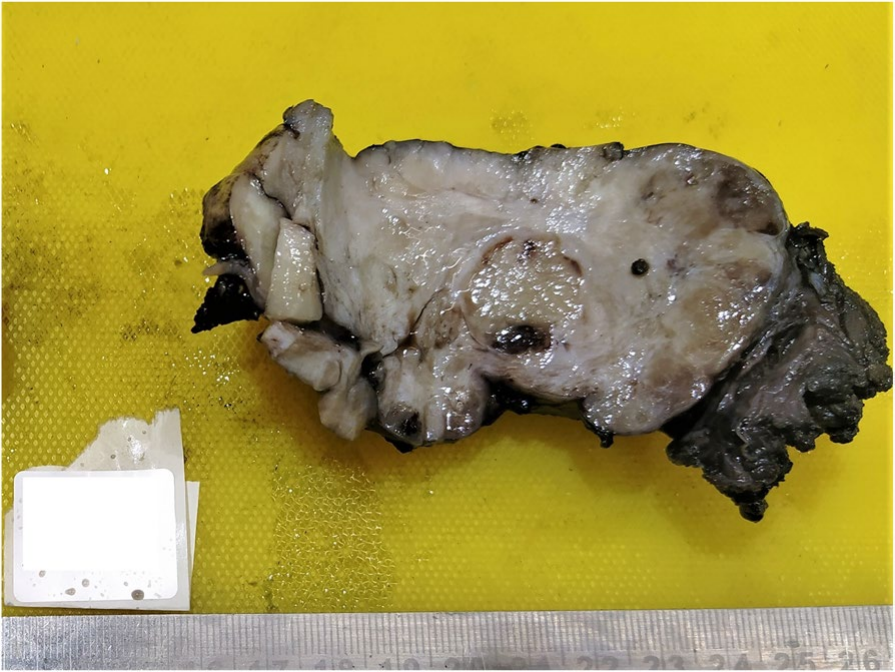
Cut section of gross specimen of penile SFT post en-bloc excision

**Fig. 4:**
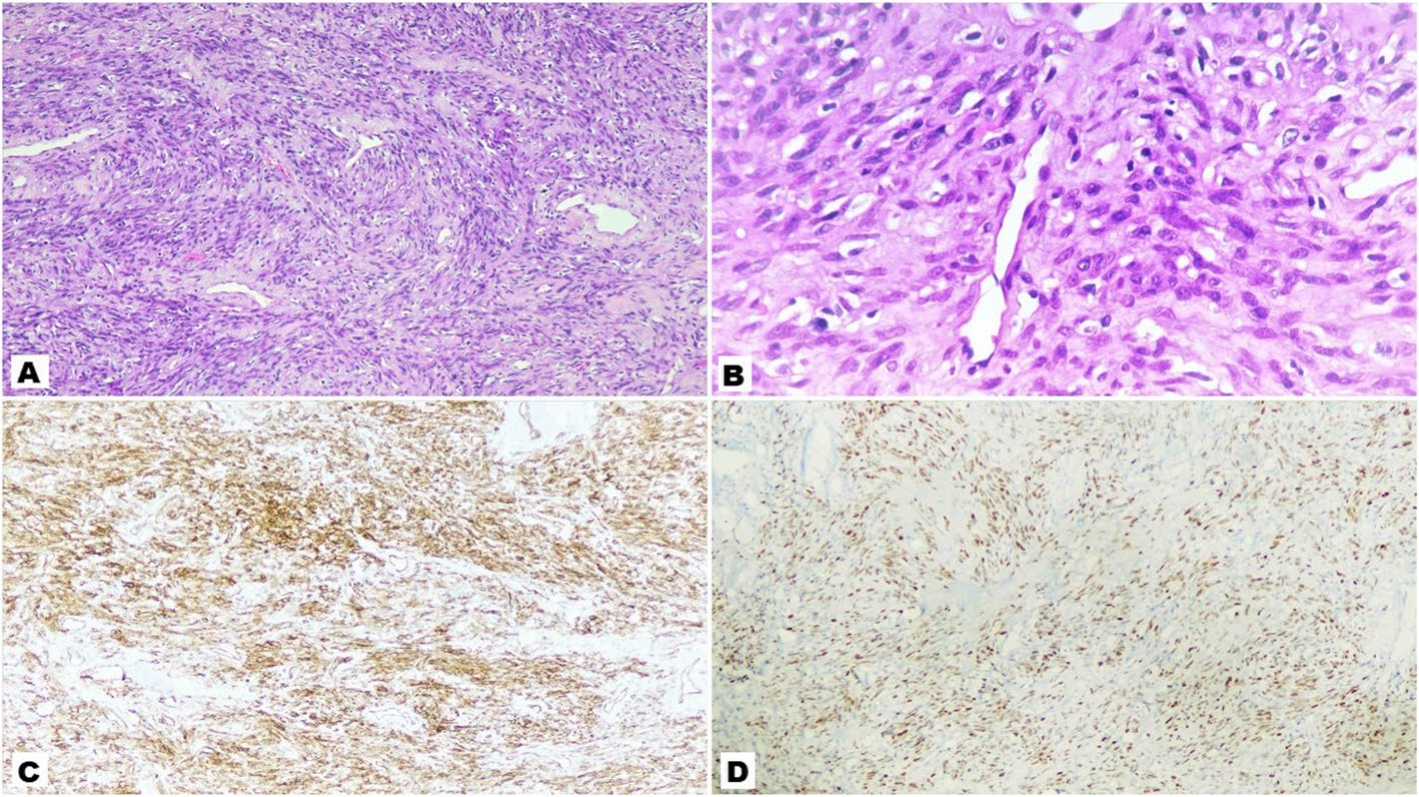
Histologic appearance of SFT. **A** 10x view H&E stain showing typical hyper and hypocellular areas in a sclerotic background, **B** 40x view of the same, **C** & **D** IHC showing tumor cells positive for CD34 and STAT6

Histopathology examination of resected enbloc specimen revealed gross features of an encapsulated and bosellated mass measuring 10 × 4.5x4cm with grey white appearing cut section which was lobulated and firm (Fig. 3). Microscopic examination showed spindle cell tumor embedded in myxohyaline stroma along with hyalinized vascular channels and inconspicuous mitotic figures with focal area of necrosis (Fig. 4A & B). On IHC tumor cells were positive for CD34 and STAT6 while negative for AE1/E3, S-100 and beta-catenin. Ki-67 proliferation index was 5% in the highest proliferating area (Fig. 4C & D).

## Discussion and conclusions

### Incidence and clinical presentation

These slow growing tumors of mesenchymal origin are now being identified with great certainty due to advancements in histology and molecular genetics. Although, most commonly presenting as asymptomatic masses identified on imaging done for other reasons, symptoms appear when large lesions cause mechanical pressure effects. Cubuk et al [23] reported a 55 year old gentlemen with a 4 × 4 cm penile SFT which was hindering sexual intercourse. At the Memorial Sloan Kettering Cancer Center (MSKCC), amongst the 4000 cases of soft tissue tumors treated over a 18-months period (1999–2001), only 79 could be identified as SFT’s [3].

### Imaging and histopathological features

Vallat-Decouveleare et al. [14] suggested that nuclear atypia, areas of increased cellularity, necrosis and ≥ 4 mitoses/10 HPF were predictive for clinical malignant behaviour and found local or distant relapse in 80% such cases, but also reported a case of clinically malignant behaviour of a histological benign appearing case. Recurrent tumor specimens showed a higher grade of atypia than the primary tumor but usually retained their immunohistochemical profile. In the study by Gold et al. [3] all the pathologic variables (mitosis, nuclear pleomorphism, cellularity, necrosis, and the presence of a malignant component) correlated with either local recurrence or metastasis and even correlated highly with one another, also, positive surgical resection margins and primary tumor sizes of ≥ 10 cm positively correlated with unfavourable clinical outcome. Lastly, the presence of a malignant component in a SFT was associated with both, worse local recurrence-free survival and metastasis-free survival (P < 0.01). Certain SFT variants such as, lipomatous variant of SFT (“lipomatous hemangiopericytoma”) showing mature fat component intermingled with typical areas of SFT and myxoid SFT have also been described and can pose a challenge for the histopathologist when establishing the diagnosis. Histopathologic spectrum encompassing branching, ectatic, hemangiopericytoma-like blood vessels, the “patternless- pattern” with or without fasicular, storiform, neural-type, herringbone and diffuse sclerosing patterns are also quite frequently identified in other, predominantly malignant, soft tissue tumors such as synovial sarcomas, malignant peripheral nerve sheath tumors, dermatofibrosarcoma protuberance (DFSP), leiomyosarcomas and liposarcomas and hence can confound the diagnosis [24]. Therefore, it is evident that an experienced soft tissue pathologist should evaluate the specimens. Positron emission tomography (PET) may be helpful to distinguish between a malignant and a benign variant of the tumor, but the gold standard for diagnosis remains incisional biopsy.

### Management and follow-up

En-bloc excision of SFT’s remains the treatment of choice. Advanced reconstructive surgical techniques allow for complete excision of large tumors with limb salvage wherever possible. Adjuvant chemotherapy or radiation therapy has been used in certain circumstances such as positive surgical margins and malignant variants, however the long term benefits remain unknown [3, 14]. Local recurrence with or without metastasis has been reported in SFTs with lung and liver being the most common site for metastasis apart from other sites such as bones, mediastinum, retroperitoneum, omentum and mesentry and patients with extra-thoracic SFT are more likely to develop metastases [25]. Demicco et al. [26] reported overall 5- and 10-year metastasis-free rates of 74% and 55%, respectively, while 5- and 10-year disease-specific survival rates were 89% and 73%. Their study proposed a risk assessment model based on age, size and mitotic index wherein patient age, tumor size, and mitotic index predicted both time to metastasis and disease-specific mortality, while necrosis predicted metastasis only. Tumor relapse can occur anywhere between 1–6 years, McMaster et al. [27] reported 10% while Vallat-Decouvelaere et al. [14] reported 40% metastases occurring at 5 event-free year. Overall patients without a histologically malignant component and with a tumor size < 10 cm in dimension can expect a favorable outcome and are adequately treated by surgery alone, and those having both a histologically malignant component and a tumor > 10 cm fare rather poorly. Optimal adjuvant treatment for the latter group of patients is not known, however, a stringent long-term follow-up is a bare minimum necessity.

Penile SFT’s can be safely removed en-bloc without significant aesthetic and functional impairment with timely intervention in a well-informed patient. The ambiguous nature of histopathological features of SFT warrant its examination with experience pathologists having special interest in soft tissue sarcomas. Ongoing research, pooled data analysis and advances in histologic, molecular and genetic techniques will allow more precise identification and categorization of these tumors, develop targeted therapy and predict future clinical course of the illness.

## Data Availability

Data sharing is not applicable to this article as no datasets were generated or analysed during the current study.

## Abbreviations

SFT: Solitary Fibrous Tumor
IHC: Immunohistochemistry
CT: Computerized Tommography
MRI: Magnetic Resonance Imaging
CD: Cluster of differentiation
IIEF: International Index of Erectile Function
FDG: Fluorodeoxyglucose
